# Evaluation of the anti-osteoarthritic effects and mechanisms of *Cissus quadrangularis* extract containing quercetin and isorhamnetin in a rat model of monosodium iodoacetate-induced osteoarthritis

**DOI:** 10.29219/fnr.v69.12173

**Published:** 2025-04-15

**Authors:** Yean-Jung Choi, Jae In Jung, Jaewoo Bae, Jae Kyoung Lee, Eun Ji Kim

**Affiliations:** 1 Department of Food and Nutrition, Sahmyook University, Seoul, Republic of Korea; 2 Industry Coupled Cooperation Center for Bio Healthcare Materials, Hallym University, Chuncheon, Republic of Korea; 3 FMCG-Korea Research Institute, FMCG-Korea Co. Ltd., Goyang, Republic of Korea

**Keywords:** Osteoarthritis, Cissus quadrangularis, quercetin, isorhamnetin, inflammation, cartilage protection, matrix metalloproteinases

## Abstract

**Background:**

Osteoarthritis (OA) is a degenerative joint disease characterized by cartilage degradation, subchondral bone erosion, and chronic inflammation. Current treatments primarily focus on symptom relief and have significant side effects, highlighting the need for safer, more effective alternatives. *Cissus quadrangularis* extract (CQE), containing bioactive flavonoids quercetin and isorhamnetin, has shown potential anti-inflammatory and cartilage-protective properties.

**Objective:**

This study aimed to investigate the anti-osteoarthritic effects and mechanisms of action of CQE in a monosodium iodoacetate (MIA)-induced OA rat model.

**Design:**

Sprague-Dawley (SD) rats were induced with OA through intra-articular injection of MIA and treated with CQE at doses of 30, 50, and 100 mg/kg body weight (BW)/day. The effects of CQE on knee joint damage, subchondral bone erosion, cartilage structure, proteoglycan content, and the expression of inflammatory mediators and matrix metalloproteinases (MMPs) were assessed using micro-computed tomography (micro-CT), histological staining, immunofluorescence, and real-time reverse transcription-polymerase chain reaction (RT-PCR).

**Results:**

CQE significantly mitigated knee joint damage, reduced subchondral bone erosion, and enhanced bone volume and trabecular structure in MIA-induced OA rats. It also preserved cartilage integrity by maintaining proteoglycan content and the expression of collagen type II alpha 1 (COL2A1) and aggrecan. Moreover, CQE suppressed the mRNA expression of inflammatory mediators [inducible nitric oxide synthase (iNOS), cyclooxygenase-2 (COX-2), and 5-lipoxygenase (5-LOX)], pro-inflammatory cytokines [interleukin (IL)-1β, IL-6, and tumor necrosis factor-α (TNF-α)], and MMPs (MMP-2, MMP-3, MMP-9, and MMP-13), indicating strong anti-inflammatory and cartilage-protective effects.

**Conclusions:**

CQE exhibits significant therapeutic potential in managing OA by targeting multiple aspects of disease progression, including inflammation, cartilage degradation, and bone erosion. Further research is needed to explore long-term efficacy, safety, and the molecular mechanisms of CQE, as well as to validate these findings in human clinical trials.

## Popular scientific summary

- *Cissus quadrangularis* extract mitigated knee joint damage, reduced subchondral bone erosion, and enhanced bone volume and trabecular structure in monosodium iodoacetate-induced osteoarthritis SD rats.
- *Cissus quadrangularis* extract suppressed the mRNA expression of inflammatory mediators, pro-inflammatory cytokines, and matrix metalloproteinases in the synovia of SD rats with osteoarthritis.
- *Cissus quadrangularis* extract exhibited therapeutic potential in managing osteoarthritis by targeting multiple aspects of disease progression.

Osteoarthritis (OA) is a prevalent chronic degenerative joint disease that is characterized by degradation and loss of articular cartilage and other joint tissues (ligaments and muscles), and chronic low-grade inflammation in the joint, which eventually leads to pain, stiffness, reduced function, and disability (1). The incidence of OA is steeply increasing due to aging (2), and as a result, the burden of OA is steadily increasing worldwide (1). Therefore, OA is considered one of the serious diseases that threaten a healthy life, especially in old age.

As OA has a multifactorial etiology, it is difficult to cure completely this disease. Currently, most of the medications available for OA are non-steroidal anti-inflammatory drugs and glucocorticoids. These medications relieve only OA pain but disregard the progression or modification of OA (3). Their extended use causes severe side effects such as bleeding and gastric ulceration (4, 5). Therefore, long-term, safe medications for the treatment of OA are imperative.

*Cissus quadrangularis* is a succulent vining plant belonging to the Vitaceae family, prominently distributed in India and Africa. It has been used as both a food item and folk medicine for more than a century. Traditionally, *C. quadrangularis* has been used to manage bone-related diseases such as fracture, pain, osteoporosis, and rheumatoid and to treat hemorrhage, dyspepsia, colic, chronic ulcer, convulsion, and helminthiasis (6–8). Based on several studies to date (9–13), *C. quadrangularis* has been used as a functional food ingredient to manage obesity and complications associated with metabolic syndrome, which is safe and free from adverse effects at the doses commonly used (14, 15).

Recently, several studies have addressed the anti-inflammatory effect of *Cissus quadrangularis* extract (CQE) (16–19). Bhujade et al. (16) reported that *C. quadrangularis* extract acted as an inhibitor of cyclooxygenase-2 (COX-2), 5-lipoxygenase (5-LOX), and proinflammatory mediators. *C. quadrangularis* extract inhibited interleukin (IL)-1β-induced inflammatory response on chondrocytes (17) and attenuated inflammatory response in ethyl phenylpropionate-induced ear edema and phlogistic agent-induced paw edema (18). In addition, CQE inhibited inflammatory response in the formaldehyde-induced and adjuvant-induced arthritis rats (19). These studies hypothesized that CQE could relieve OA by inhibiting inflammatory response because inflammation in the joint is one of the etiologies of OA. Lakshmanan et al. (20) reported that the ethanol extract of *C. quadrangularis* stems attenuated OA progression in monosodium iodoacetate (MIA)-induced OA rats. Although this study has exhibited the anti-OA effect of *C. quadrangularis,* it is insufficient to explain the mechanisms by which it attenuates OA progression. Therefore, more studies are needed to confirm the anti-OA effect of *C. quadrangularis* and to elucidate its molecular mechanisms of action.

In this study, we aimed to evaluate the anti-osteoarthritic effects of a standardized CQE containing quercetin (12.2 mg/g) and isorhamnetin (0.37 mg/g) in an MIA-induced OA model in Sprague-Dawley (SD) rats. Additionally, we sought to investigate the underlying mechanisms contributing to its therapeutic effects. Our findings suggest that CQE may exert significant anti-osteoarthritic activity by reducing inflammation, preserving cartilage integrity, and protecting against bone degradation, offering a potential multifaceted approach to managing OA.

## Materials and methods

### Materials

The following materials were procured from the indicated suppliers: MIA, hematoxylin and eosin (H&E), and 4’,6-diamidino-2-phenylindole (DAPI) from Sigma-Aldrich Co. (St. Louis, MO, USA); anti-aggrecan and anti-COL2A1 antibody from Santa Cruz Biotechnology (Santa Cruz, CA, USA); fluorochrome-conjugated secondary antibodies (Alexa-488 and 564) from Thermo Fisher Scientific (Waltham, MA, USA); safranin O from ScienCell Research Laboratories (Carlsbad, CA, USA); TRIzol reagent from Invitrogen Life Technologies (Carlsbad, CA, USA); HyperScript^TM^ RT master mix from GeneAll Biotechnology (Seoul, Korea); QuantiNova SYBR Green PCR kit from Qiagen (Valencia, CA, USA).

### Preparation of CQE

The CQE, marketed as CISSUSLEAN®, was generously provided by StarHiHerbs Private Limited (Bengaluru, India). To prepare the extract, dried stems of *C. quadrangularis* were subjected to extraction with water at 100°C for 3 h. The resulting extract was then filtered and concentrated through vacuum evaporation. The filtrate was further dried using a spray dryer to reduce the moisture content to less than 5%, yielding the final powdered CQE. For standardization, the contents of the bioactive compounds quercetin and isorhamnetin in CQE were quantified using high-performance liquid chromatography (HPLC). The standardized extract was found to contain 12.2 mg/g of quercetin and 0.37 mg/g of isorhamnetin, ensuring consistency and potency in the prepared CQE.

### Ethical statement and animal care

All animal experimental protocols applied to this study received approval from the Institutional Animal Care and Use Committee of Hallym University (approved number: Hallym 2023-46). All animal experiments followed the guidelines for the care and use of laboratory animals.

Specific pathogen-free, 6-week-old, male SD rats were procured from Dooyeol Biotech Co. Ltd. (Seoul, Korea). They were kept at the animal facility of Hallym University, which was maintained at 23 ± 3°C and 50 ± 10% relative humidity, with a 12-h light/dark cycle. The rats had *ad libitum* access to a commercial non-purified rodent diet and tap water.

### Treatment and induction of OA

After acclimation for 1 week, the SD rats were randomly assorted into six groups (*n* = 10 rats/group) as follows: (1) Normal control group (NC), (2) OA control group (OC), (3) OA + 30 mg/kg body weight (BW)/day CQE-treated group (O+C30), (4) OA + 50 mg/kg BW/day CQE-treated group (O+C50), (5) OA + 100 mg/kg BW/day CQE-treated group (O+C100), and (6) OA + 150 mg/kg BW/day methyl sulfonyl methane (MSM)-treated group (O+M). The rats in each group received oral administration of either CQE or MSM (positive control), which dissolved in sterile water, daily for 5 weeks. CQE or MSM was administered once daily to ensure consistent dosing and experimental conditions. We chose MSM as our study’s positive control because of its well-established ability to reduce cartilage loss and inflammation, which are indicative of OA (21, 22). The rats in the NC and OC groups were administered an equal volume of sterile water as a vehicle by oral gavage. Two weeks after CQE or MSM administration, all rats, excluding the NC group, received an intra-articular injection of 3 mg of MIA in 50 μL saline into the right knee to induce OA, as described previously (23, 24). The rats in the NC group were injected with 50 μL saline instead of MIA solution. At the end of the experiment, the rats were euthanized by carbon dioxide asphyxiation, after which the knee joints and the synovium were immediately harvested for further analysis.

### Micro-computed tomography (micro-CT) analysis

To investigate changes in the knee joint micro-architecture, micro-CT analysis was conducted using a micro-CT scanner and image analysis software (VivaCT 80, Scanto Medical AG, Brűttisellen, Switzerland) at Metropolitan Seoul Center of the Korea Basic Science Institute, as described previously (24). The femorotibial joint underwent scanning, the results of which were reconstructed into three-dimensional or two-dimensional images for three-dimensional or two-dimensional morphometric parameters analysis. Within the femorotibial joint, bone surface/bone volume (BS/BV, %) of the subchondral bone was evaluated to determine the severity of bone erosion. Additionally, in the tibia, parameters such as bone volume fraction (bone volume/total volume, BV/TV, %), trabecular number (Tb.N, 1/mm), and trabecular thickness (Tb.Th, mm) of the metaphysis were analyzed to discern structural alterations.

### Histological analysis

The knee joints were fixed in 4% paraformaldehyde, decalcified in Calci Clear Rapid (National Diagnostics, Atlanta, GA, USA), and embedded in paraffin. Subsequently, the tissues were cut into 5 μm-thick sections and stained with H&E and safranin O according to the manufacturer’s instructions. The stained tissues were scrutinized under a microscope (AxioImager, Carl Zeiss, Jena, Germany). The randomly selected areas were photographed at 100x magnification and blindly examined. The severity of knee OA was assessed according to the Osteoarthritis Research Society International (OARSI) scoring system (25).

### Immunofluorescence (IF) staining

Sections of paraffin-embedded knee joint tissues, 5 μm thick, were deparaffinized and blocked with 5% bovine serum albumin. IF staining was performed using antibodies against COL2A1 and aggrecan, along with fluorochrome-conjugated secondary antibodies (Alexa-488 or 564). The nuclei were counterstained with DAPI. The stained tissues were observed under a microscope (AxioImager, Carl Zeiss), with randomly selected areas photographed at 400x magnification and examined blindly. The immune-positive cells were quantified with Image M1 Software (Carl Zeiss).

### Real-time reverse transcription-polymerase chain reaction

Real-time reverse transcription-polymerase chain reaction (RT-PCR) was conducted as described previously (24). Total RNA from the synovium of the knee joint was extracted using a TRIzol reagent. Single-strand complementary DNA was synthesized using the HyperScript^TM^ RT master mix kit, and real-time PCR was conducted employing the QuantiNova SYBR Green PCR kit (Qiagen) and Rotor-Gene 3,000 instrument (Corbett Research, Mortlake, Australia). The sequences of the primers used in this study are shown in Table 1. Data analysis was performed using the Rotor-Gene 6,000 Series system software program, version 6 (Corbett Research). The relative mRNA expression levels of target genes were normalized to those of glyceraldehyde 3-phosphate dehydrogenase (GAPDH).

**Table 1:** Primer sequences used in this study

Target gene	Forward primer (5’-3’)	Reverse primer (5’-3’)
*5-Lox*	CCATCCAGCTCAACCAAACC	GATGTGTGCGGAGAAGATGG
*Cox-2*	TGCGATGCTCTT CCGAGCTGTGCT	TCAGGAAGTTCCTTATTTCCTTTC
*Il-1β*	CACCTCTCAAGCAGAGCACAG	GGGTTCCATGGTGAAGTCAAC
*Il-6*	TCCTACCCCAACTTCCAATGCTC	TTGGATGGTCTTGGTCCTTAGCC
*iNos*	CACCACCCTCCTTGTTCAAC	CAATCCACAACTCGCTCCAA
*Mmp-2*	TGGGGGAGATTCTCACTTTG	CCATCAGCGTTCCCATACTT
*Mmp-3*	TGGGAAGCCAGTGGAAATG	CCATGCAATGGGTAGGATGAG
*Mmp-9*	TGCTCCTGGCTCTAGGCTAC	TTGGAGGTTTTCAGGTCTCG
*Mmp-13*	TGGCGACAAAGTAGATGCTG	TGGCATGACTCTCACAATGC
*Tnf-α*	AAATGGGCTCCCTCTCATCAGTTC	TCTGCTTGGTGGTTTGCTACGAC
*Gapdh*	CTCAACTACATGGTCTACATGTTCCA	CTTCCCATTCTCAGCCTTGACT

### Statistical analysis

All data were expressed as the mean ± standard error of the mean (SEM). Statistical analyses were performed using Student’s *t*-test and one-way analysis of variance (ANOVA) to evaluate the significance of differences between groups. When ANOVA indicated significant differences, Duncan’s multiple range test was used for post hoc comparisons to identify specific differences between group means. A *p*-value of less than 0.05 was considered statistically significant. All statistical analyses were conducted using the Statistical Analysis System, Windows version 9.4 (SAS Institute, Cary, NC, USA).

## Results

### CQE mitigates MIA-induced knee joint damage in OA rats

**Fig. 1:**
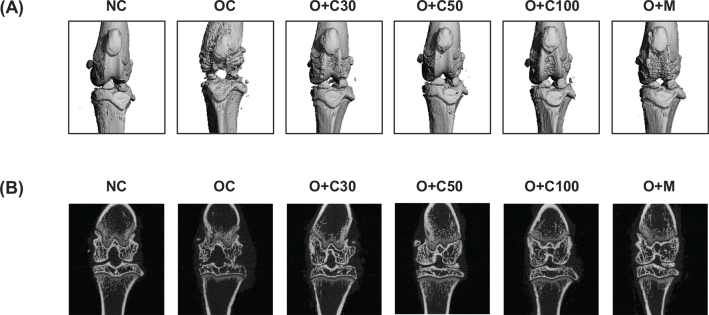
Effect of CQE on cartilage degradation in SD rats with MIA-induced osteoarthritis. SD rats were orally administered CQE at doses of 30, 50, or 100 mg/kg BW/day or MSM at 150 mg/kg BW/day, over a period of 5 weeks. Fourteen days after the initiation of CQE or MSM administration, MIA was injected into the intra-articular joint of the right knee to induce osteoarthritis. Measurements and sample harvesting were conducted 3 weeks post-MIA injection, specifically on day 21, to evaluate the effects of the treatments. The femorotibial joint was analyzed using micro-CT to assess joint damage and cartilage degradation. (A) Representative three-dimensional micro-CT images (*n* = 5). (B) Representative two-dimensional micro-CT images (*n* = 5).

Micro-CT analysis was conducted on day 21, 3 weeks post-MIA injection, to investigate the effect of CQE on the femorotibial joint, particularly focusing on the epiphysis of the proximal tibia. Significant knee joint damage was observed in the OC group compared to the NC group, as shown in Fig. 1A and 1B. However, treatment with CQE (30, 50, and 100 mg/kg BW/day) and MSM significantly mitigated the MIA-induced knee joint damage. Specifically, the O+C30, O+C50, O+C100, and O+M groups showed a marked reduction in knee joint damage compared to the OC group.

### CQE reduces subchondral bone erosion in MIA-induced OA rats

**Fig. 2:**
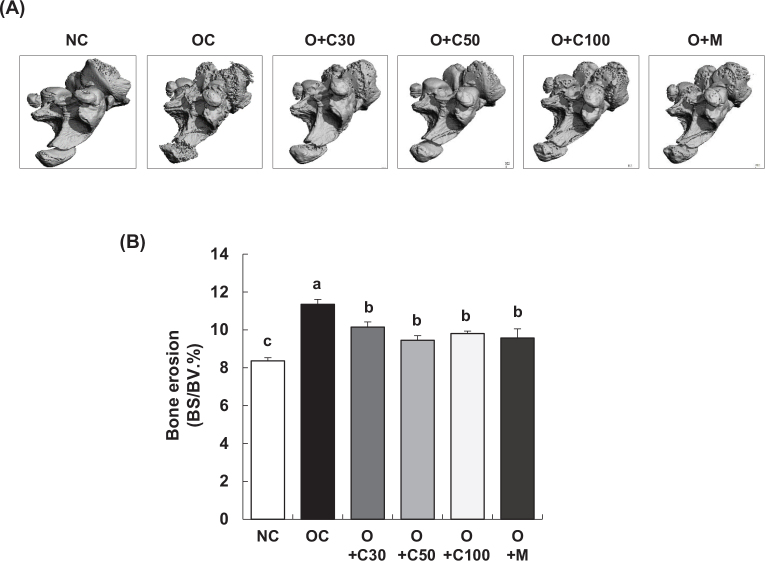
Effect of CQE on subchondral bone integrity in SD rats with MIA-induced osteoarthritis. SD rats were administered CQE and injected with MIA as described in Figure 1. Measurements and sample harvesting were conducted 3 weeks post-MIA injection, specifically on day 21, to evaluate the effects of the treatments on subchondral bone integrity. The femorotibial joint was analyzed using micro-CT. (A) Gray-scale reconstructed images of the subchondral bone in the femorotibial joint (*n* = 5). (B) Bone surface/bone volume ratio (BS/BV) of the subchondral bone in the femorotibial joint (*n* = 5). Each bar represents the mean ± SEM (*n* = 5). Statistical significance was evaluated using Student’s *t*-test and one-way analysis of variance (ANOVA). Different lowercase letters indicate statistically significant differences between groups, as determined by Duncan’s test (*p* < 0.05).

As shown in Fig. 2A and 2B, the bone surface/bone volume ratio (BS/BV, %) of the subchondral bone, which indicates the extent of knee joint erosion, was significantly higher in the OC group compared to the NC group (*p* < 0.001). However, treatment with CQE at doses of 30, 50, and 100 mg/kg BW/day, as well as MSM, resulted in a significant reduction in the BS/BV ratio in the O+C30, O+C50, O+C100, and O+M groups compared to the OC group (*p* < 0.05).

### CQE enhances bone volume and trabecular structure in MIA-induced OA rats

**Fig. 3:**
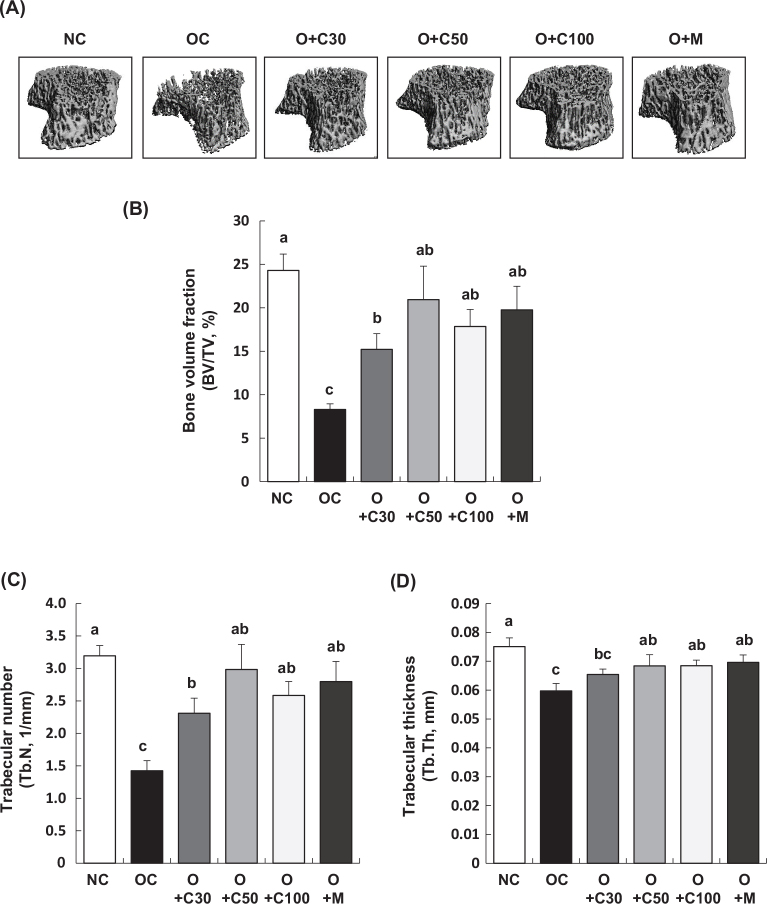
Effect of CQE on bone volume and trabecular structure in SD rats with MIA-induced osteoarthritis. SD rats were administered CQE and injected with MIA as described in Figure 1. Measurements and sample harvesting were conducted 3 weeks post-MIA injection, specifically on day 21, to assess the effects of the treatments on bone volume and trabecular structure. The femorotibial joint was analyzed using micro-CT. (A) Gray-scale reconstructed images of the metaphysis in the tibia. (B) Bone volume/total volume ratio (BV/TV), (C) Trabecular number (Tb.N), and (D) Trabecular thickness (Tb.Th) of the metaphysis in the tibia (*n* = 5). Each bar represents the mean ± SEM (*n* = 5). Statistical significance was evaluated using Student’s *t*-test and one-way analysis of variance (ANOVA). Different lowercase letters indicate statistically significant differences between groups, as determined by Duncan’s test (*p* < 0.05).

Figure 3A and 3B-3D show that the bone volume fraction (bone volume/total volume ratio, BV/TV, %), which represents the proportion of cancellous bone within the volume of interest, as well as the trabecular number (Tb.N, 1/mm), reflecting the number of cancellous bones per unit length, and trabecular thickness (Tb.Th, mm), were all significantly lower in the OC group compared to the NC group (*p* < 0.001). However, treatment with CQE at doses of 30, 50, and 100 mg/kg BW/day, as well as MSM, resulted in a significant increase in the BV/TV ratio and Tb.N in the O+C30, O+C50, O+C100, and O+M groups compared to the OC group, indicating a protective effect against bone structural changes due to OA (*p* < 0.05). Additionally, Tb.Th was significantly higher in the O+C50, O+C100, and O+M groups compared to the OC group.

### CQE alleviates histological and proteoglycan loss in knee cartilage of MIA-induced OA rats

**Fig. 4:**
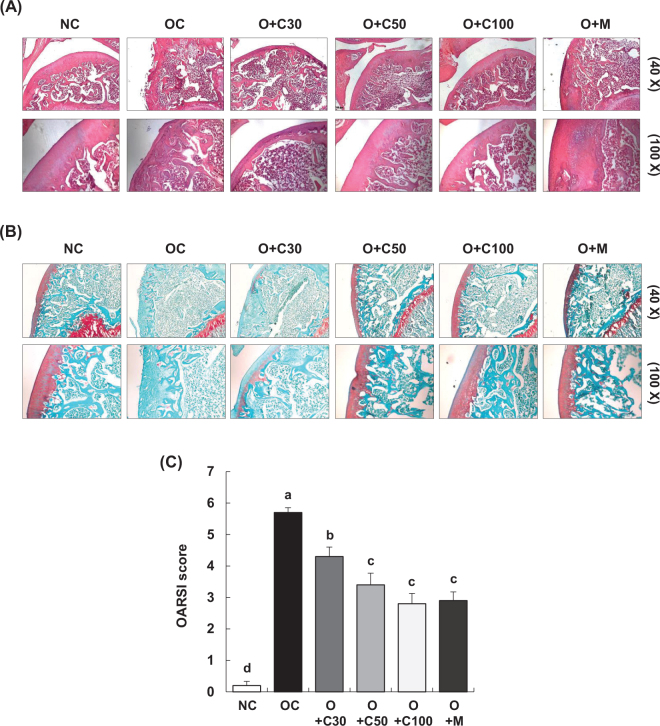
Effect of CQE on histological changes in the articular cartilage of SD rats with MIA-induced osteoarthritis. SD rats were administered CQE and injected with MIA as described in Figure 1. Measurements and sample harvesting were conducted 3 weeks post-MIA injection, specifically on day 21, to evaluate the effects of the treatments on articular cartilage. The cartilage was stained with (A) hematoxylin and eosin (H&E) and (B) safranin O and was subjected to Osteoarthritis Research Society International (OARSI) scoring. Representative staining images are shown (*n* = 5), with a magnification of 100×. (C) OARSI scores for cartilage degradation (*n* = 5). Each bar represents the mean ± SEM (*n* = 5). Statistical significance was evaluated using Student’s *t*-test and one-way analysis of variance (ANOVA). Different lowercase letters indicate statistically significant differences between groups, as determined by Duncan’s test (*p* < 0.05).

Histomorphological changes in knee joint cartilage were assessed through H&E staining, conducted on day 21, 3 weeks post-MIA injection, as shown in Fig. 4A. The articular cartilage in the NC group remained smooth and well preserved, with no signs of deformation or damage. In contrast, the OC group exhibited deformed and damaged cartilage with a worn surface. Treatment with CQE and MSM, particularly in the O+C50, O+C100, and O+M groups, significantly mitigated the cartilage damage, bringing the condition closer to that of the NC group.

Proteoglycan content in the cartilage tissue was evaluated using safranin O staining, with results also taken on day 21 post-MIA injection, as presented in Fig. 4B. In the NC group, the cartilage maintained a normal structure with uniform proteoglycan distribution and no evidence of tissue deformation or loss. Conversely, the OC group displayed severely damaged cartilage and a substantial loss of proteoglycan, which was absent from the cartilage surface. The O+C50, O+C100, and O+M groups showed significant improvement, with reduced cartilage damage and less proteoglycan loss compared to the OC group. The effects were more pronounced in the O+C50 and O+C100 groups than in the O+C30 group.

The OARSI score, which quantifies the severity of knee OA, was significantly higher in the OC group compared to the NC group (*p* < 0.001). However, the OARSI scores were significantly reduced in the O+C30, O+C50, O+C100, and O+M groups compared to the OC group, with measurements taken 3 weeks post-MIA injection, specifically on day 21 (Fig. 4C).

### CQE preserves COL2A1 and aggrecan expression in articular cartilage of MIA-induced OA rats

**Fig. 5:**
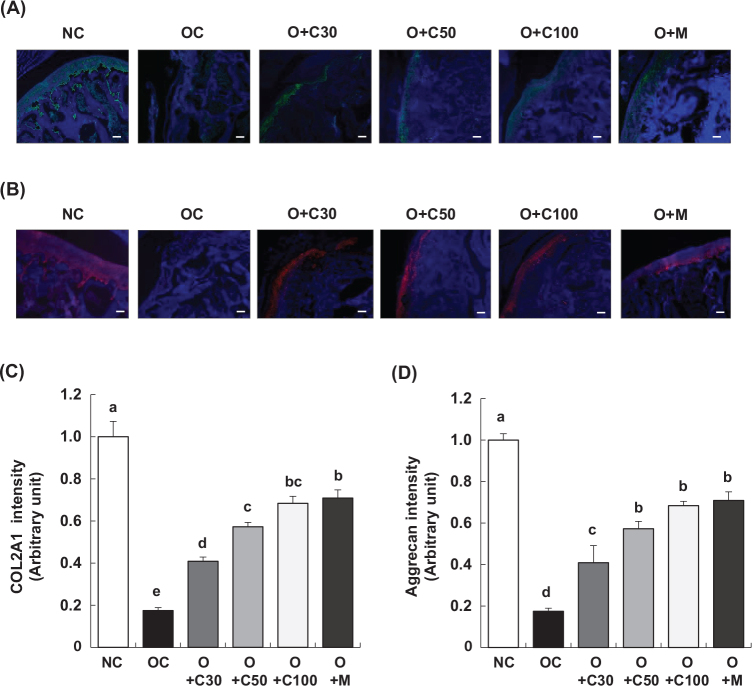
Effect of CQE on the expression of COL2A1 and aggrecan in the articular cartilage of SD rats with MIA-induced osteoarthritis. SD rats were administered CQE and injected with MIA as described in Figure 1. Measurements and sample harvesting were conducted 3 weeks post-MIA injection, specifically on day 21, to evaluate the effects of the treatments on the expression of key cartilage components. Articular cartilage was stained with antibodies against (A) COL2A1 and (B) aggrecan. Representative staining images are shown (*n* = 5), with a magnification of 100×. (C, D) The staining intensity of the indicated proteins was quantified. Each bar represents the mean ± SEM (*n* = 5). Statistical significance was evaluated using Student’s *t*-test and one-way analysis of variance (ANOVA). Different lowercase letters indicate statistically significant differences between groups, as determined by Duncan’s test (*p* < 0.05).

Immunofluorescence (IF) staining, performed on day 21, 3 weeks post-MIA injection, was used to assess the expression of collagen type II alpha 1 (COL2A1) and aggrecan in the articular cartilage tissue. As shown in Fig. 5A and 5B, there was a significant decrease in the expression of COL2A1 and aggrecan in the OC group compared to the NC group. However, the O+C30, O+C50, O+C100, and O+M groups displayed relatively higher levels of COL2A1 and aggrecan expression compared to the OC group, suggesting a reduction in the extent of downregulation associated with OA (Fig. 5C and 5D).

### CQE reduces the mRNA expression of inflammatory mediators and cytokines in MIA-induced OA rats

**Fig. 6:**
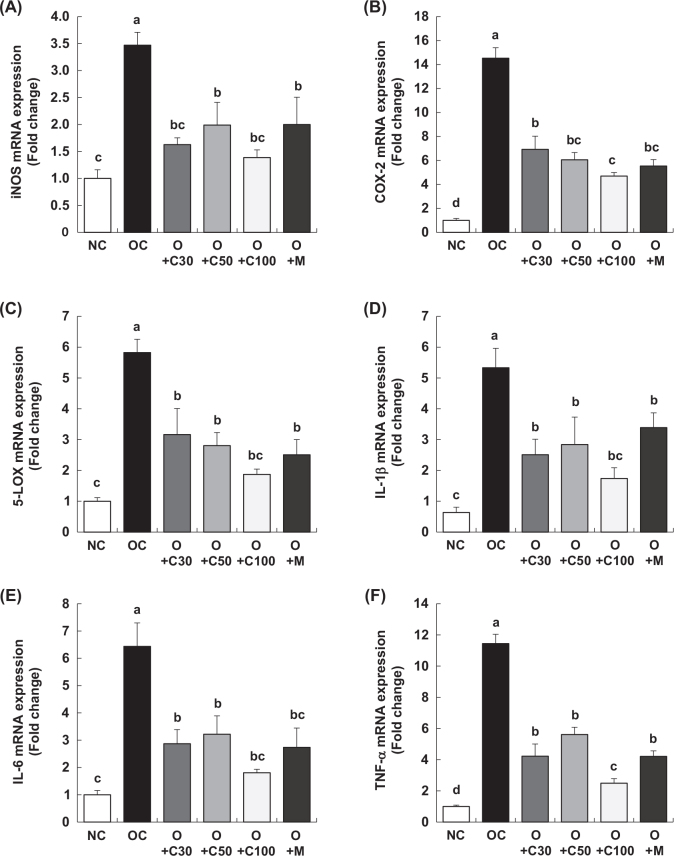
Effect of CQE on mRNA expression of inflammatory mediators and cytokines in the synovia of SD rats with MIA-induced osteoarthritis. SD rats were administered CQE and injected with MIA as described in Figure 1. Measurements and sample harvesting were conducted 3 weeks post-MIA injection, specifically on day 21, to assess the effects of the treatments on inflammatory mediators and cytokines. Total RNA was extracted from the synovia, followed by reverse transcription and real-time PCR analysis. mRNA expression levels of (A) iNOS, (B) COX-2, (C) 5-LOX, (D) IL-1β, (E) IL-6, and (F) TNF-α were normalized to GAPDH and expressed relative to the NC group. Each bar represents the mean ± SEM (*n* = 5). Statistical significance was evaluated using Student’s *t*-test and one-way analysis of variance (ANOVA). Different lowercase letters indicate statistically significant differences between groups, as determined by Duncan’s test (*p* < 0.05).

Figure 6 shows the results of mRNA expression analysis for inducible nitric oxide synthase (iNOS), cyclooxygenase-2 (COX-2), and 5-lipoxygenase (5-LOX), which are enzymes responsible for the synthesis of inflammatory mediators NO, PGE2, and LTB4, respectively. These analyses were performed on samples collected on day 21, 3 weeks post-MIA injection, after extracting total RNA from the knee joint synovium and conducting real-time RT-PCR. Figure 6A demonstrates that iNOS mRNA expression was significantly upregulated in the OC group compared to the NC group (*p* < 0.001). In contrast, iNOS mRNA levels were significantly reduced in the O+C30, O+C50, O+C100, and O+M groups compared to the OC group (*p* < 0.05).

Similarly, as shown in Fig. 6B and 6C, COX-2 and 5-LOX mRNA levels were significantly higher in the OC group than in the NC group (*p* < 0.001). However, the groups treated with CQE and MSM (O+C30, O+C50, O+C100, and O+M) exhibited significant reductions in both COX-2 and 5-LOX mRNA expressions compared to the OC group (*p* < 0.05).

As outlined in Fig. 6D-6F, the mRNA expression levels of the inflammatory cytokines interleukin (IL)-1β, IL-6, and tumor necrosis factor-α (TNF-α) were markedly elevated in the OC group compared to the NC group. However, these levels were significantly decreased in the O+C30, O+C50, O+C100, and O+M groups when compared to the OC group (*p* < 0.05).

### CQE differentially suppresses MMPs mRNA expression in the synovium of MIA-induced OA rats

**Fig. 7:**
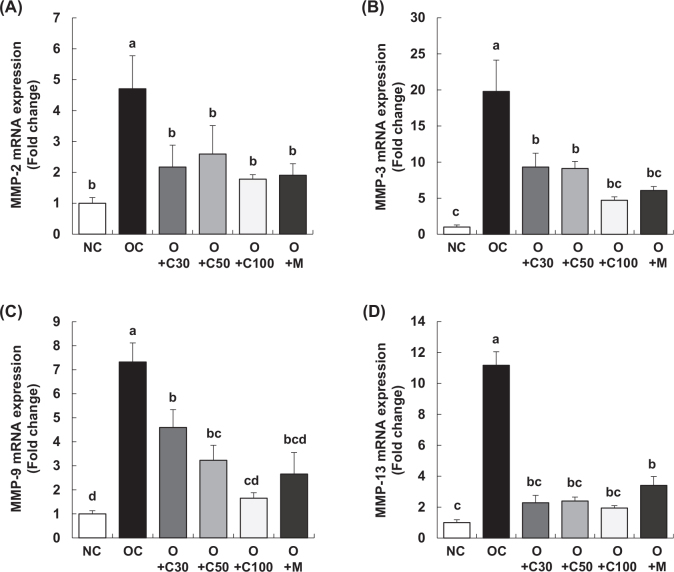
Effect of CQE on mRNA expression of MMPs in the synovia of SD rats with MIA-induced osteoarthritis. SD rats were administered CQE and injected with MIA as described in Fig. 1. Measurements and sample harvesting were conducted 3 weeks post-MIA injection, specifically on day 21, to evaluate the effects of the treatments on MMPs. Total RNA was extracted from the synovia, followed by reverse transcription and real-time PCR analysis. mRNA expression levels of (A) MMP-2, (B) MMP-3, (C) MMP-9, and (D) MMP-13 were normalized to GAPDH and expressed relative to the NC group. Each bar represents the mean ± SEM (*n* = 5). Statistical significance was evaluated using Student’s *t*-test and one-way analysis of variance (ANOVA). Different lowercase letters indicate statistically significant differences between groups, as determined by Duncan’s test (*p* < 0.05).

Figure 7 presents the mRNA expression levels of matrix metalloproteinases (MMP)-2, MMP-3, MMP-9, and MMP-13 in the knee joint synovium, with samples collected on day 21, 3 weeks post-MIA injection, to evaluate the effects of the treatments. The expression of each of these MMPs was significantly upregulated in the OC group compared to the NC group. Although there were differences in the degree of expression for each MMP, all OA test groups (O+C30, O+C50, O+C100, and O+M) exhibited a significant decrease in the mRNA expression levels of MMP-2, MMP-3, MMP-9, and MMP-13 compared to the OC group (*p* < 0.05).

## Discussion

The findings of this study provide compelling evidence that CQE enriched with quercetin and isorhamnetin exhibits significant protective effects against the progression of OA in an MIA-induced rat model. Specifically, micro-CT analysis demonstrated that CQE effectively reduced knee joint damage and subchondral bone erosion while enhancing bone volume and trabecular structure. Furthermore, histological analysis revealed that CQE alleviated cartilage damage and preserved proteoglycan content, with OARSI scores indicating a reduction in the severity of OA. Immunofluorescence staining further confirmed that CQE preserved the expression of key cartilage components such as COL2A1 and aggrecan. These outcomes suggest that CQE exerts potent anti-inflammatory and cartilage-protective effects, as evidenced by the significant reduction in the mRNA expression of inflammatory mediators, pro-inflammatory cytokines, and MMPs. Collectively, these results align with the growing body of literature supporting the anti-inflammatory and cartilage-protective properties of CQE, demonstrating its potential as a therapeutic agent for managing OA.

Our study clearly shows that CQE significantly mitigated knee joint damage and reduced subchondral bone erosion, two critical factors in the progression of OA. These protective effects were consistently observed across all tested doses of CQE, indicating a potential dose-dependent response. Moreover, the enhancement of bone volume and trabecular structure underscores CQE’s role in preserving bone integrity, which is crucial for preventing the debilitating effects of OA. The findings of our micro-CT analysis are supported by several studies that have demonstrated similar results in comparable OA models. The reduction in the bone surface/bone volume ratio and the improvement in bone structural parameters further reinforce the efficacy of CQE in protecting bone architecture and mitigating damage in OA. This effect is corroborated by studies such as Lakshmanan et al. (20) and Bhujade et al. (16), which found that CQE attenuates subchondral bone erosion and suppresses inflammatory pathways that contribute to bone degradation. Kanwar et al. (17) and Panthong et al. (18) also highlighted CQE’s ability to protect bone architecture and mitigate damage, reinforcing its role in preserving subchondral bone integrity in OA models. Moreover, CQE treatment significantly increased bone volume fraction, trabecular number, and trabecular thickness, indicating a protective effect against bone structural changes in MIA-induced OA rats. Several studies support these findings. Lakshmanan et al. (20) reported that CQE significantly improved bone volume and trabecular structure, consistent with our observations. Kothari et al. (15) also found that CQE promoted bone formation and enhanced trabecular thickness and bone density, which aligns with the improvements seen in our study. Additionally, Gupta et al. (26) and Kanwar et al. (17) confirmed the protective effects of CQE on bone volume and trabecular integrity. Collectively, these studies reinforce the efficacy of CQE in promoting bone health and preserving bone structure in OA models.

In addition to its effects on bone integrity, CQE significantly alleviated cartilage degradation and preserved proteoglycan content in the knee joints of OA rats. This was evidenced by the reduction in OARSI scores, confirming CQE’s protective effects against OA progression. At the molecular level, immunofluorescence staining revealed that CQE maintained cartilage integrity by preserving the expression of key components such as COL2A1 and aggrecan. These findings are consistent with other studies that have reported similar effects in OA models. The preservation of these cartilage components, as observed in our study, is further validated by research demonstrating CQE’s ability to inhibit the degradation of collagen and proteoglycans, thereby reinforcing its chondroprotective effects. Bhujade et al. (16) found that CQE reduced cartilage degradation and preserved proteoglycan content in an OA model, and Lakshmanan et al. (20) reported similar effects in MIA-induced OA mice. Additionally, Kumar et al. (19) confirmed that CQE preserved cartilage structure and proteoglycan levels, while Kanwar et al. (17) and Panthong et al. (18) demonstrated CQE’s anti-inflammatory and chondroprotective effects, further supporting its efficacy in reducing cartilage damage. Moreover, CQE treatment significantly preserved the expression of COL2A1 and aggrecan in the articular cartilage of MIA-induced osteoarthritic rats, counteracting the downregulation typically seen in OA. This preservation of key cartilage components is further validated by several studies, including those by Kanwar et al. (17) and Kumar et al. (19), who reported that CQE maintained the expression of collagen type II and aggrecan under inflammatory conditions. Gupta et al. (26) and Bhujade et al. (16) also demonstrated that CQE inhibited the degradation of collagen and proteoglycans, while Panthong et al. (18) emphasized CQE’s ability to protect cartilage by preserving collagen type II and aggrecan levels across various arthritis models, reinforcing the protective effects observed in our study.

This study also highlights CQE’s significant impact on the downregulation of key inflammatory mediators, including iNOS, COX-2, and 5-LOX, as well as pro-inflammatory cytokines such as IL-1β, IL-6, and TNF-α. These findings emphasize CQE’s potent anti-inflammatory properties, which likely contribute to its overall protective effects against OA. The suppression of MMPs, specifically MMP-2, MMP-3, MMP-9, and MMP-13, suggests that CQE may prevent cartilage breakdown by inhibiting enzymes critical to extracellular matrix degradation. These molecular effects are consistent with findings from other studies that have demonstrated similar outcomes in inflammatory and OA models. On a molecular level, as shown in Figs. 6 and 7, CQE treatment significantly reduced the mRNA expression of inflammatory enzymes and cytokines in the synovium of knee joints in MIA-induced OA mice. These findings are supported by several studies. For example, Bhujade et al. (16) demonstrated that CQE significantly suppressed the expression of COX-2, 5-LOX, IL-1β, and TNF-α in various inflammation models, consistent with our results. Similarly, Lakshmanan et al. (20) found that CQE effectively reduced the expression of inflammatory markers in MIA-induced OA mice. Kanwar et al. (17) and Kumar et al. (19) also reported that CQE significantly reduced the mRNA levels of IL-1β, IL-6, and TNF-α in OA models, aligning with our findings. Panthong et al. (18) further highlighted CQE’s anti-inflammatory properties, demonstrating its ability to downregulate enzymes and cytokines involved in inflammation, thereby reinforcing the anti-inflammatory effects observed in our study. Additionally, CQE treatment significantly reduced the mRNA expression levels of MMP-2, MMP-3, MMP-9, and MMP-13 in the knee joint synovium of MIA-induced OA rats. This differential inhibition of MMP expression is consistent with findings from other studies, such as Bhujade et al. (16), which showed that CQE suppressed MMP-2 and MMP-9 in an inflammatory model. Similarly, Lakshmanan et al. (20) reported that CQE helped preserve cartilage integrity by reducing MMP-3 and MMP-13 expressions. Kanwar et al. (17) and Kumar et al. (19) also observed the downregulation of MMP-9 and MMP-13 by CQE, which contributed to reduced cartilage degradation, while Panthong et al. (18) emphasized CQE’s ability to inhibit MMP activity, particularly MMP-3 and MMP-9, in inflammatory and arthritic conditions, reinforcing the protective effects observed in our study.

The inclusion of quercetin and isorhamnetin in CQE likely plays a crucial role in enhancing its therapeutic efficacy through synergistic effects. Both flavonoids are well-known for their anti-inflammatory and antioxidant properties, and their combination in CQE may amplify the extract’s overall effectiveness in managing OA. This synergistic interaction enables a multifaceted approach to targeting multiple pathways involved in OA progression, including inflammation, cartilage degradation, and bone erosion. Quercetin is recognized for its anti-inflammatory and chondroprotective effects, as it inhibits key mediators such as COX-2, TNF-α, and MMPs, all of which are crucial in the progression of OA (27). Similarly, isorhamnetin, a methylated derivative of quercetin, inhibits the NF-κB signaling pathway, reducing inflammation and MMP expression (28). The literature supports the idea that the combined use of quercetin and isorhamnetin can enhance bioavailability and efficacy, providing greater protection against OA than either compound alone. Studies support the role of quercetin and isorhamnetin in reducing cartilage degradation and inflammation in OA models (29–31). Consequently, combining these flavonoids in CQE may produce synergistic effects, targeting multiple pathways involved in OA progression to enhance overall therapeutic efficacy. This synergistic effect is further supported by literature demonstrating enhanced bioavailability and efficacy when flavonoids are combined, suggesting that the combined use of quercetin and isorhamnetin may provide greater protection against OA than either compound alone. The concentrations of quercetin (12.2 mg/g) and isorhamnetin (0.37 mg/g) used in our CQE formulation are consistent with those reported in other studies, further supporting the potential efficacy of this combination in the treatment of OA. Wang et al. (32) and Wong et al. (33) demonstrated significant anti-inflammatory and oxidative stress-reducing effects using quercetin doses ranging from 10 to 100 mg/kg in an OA model, supporting the dose used in our CQE formulation (8, 34). Additionally, Ahn et al. (35) explored isorhamnetin at a dose similar to that in our study (approximately 0.3 mg/g) and found it effective in reducing inflammation. Furthermore, Comalada et al. (36) discussed the synergistic effects of combining flavonoids such as quercetin and isorhamnetin, suggesting that their combined use at lower doses may enhance anti-inflammatory and antioxidant properties. These studies provide a strong foundation for the concentrations used in our study and support the potential efficacy of CQE in the treatment of OA.

Despite the promising results, this study has several limitations that should be considered. The use of a rat model of MIA-induced OA, although well established, may not fully capture the complexity of human OA, which could limit the direct applicability of these findings to clinical settings. Moreover, the study was conducted over a relatively short period, leaving the long-term effects of CQE unexplored. Additionally, only a specific dose of CQE was tested, without assessing a broader range or determining the optimal dose. While the study demonstrated beneficial effects on inflammation, cartilage preservation, and MMP expression, it did not delve deeply into the molecular mechanisms underlying these effects. Furthermore, the study did not compare CQE to standard OA treatments, such as NSAIDs or corticosteroids, limiting the scope of its efficacy evaluation. Lastly, the absence of human clinical data underscores the need for clinical trials to confirm the safety and efficacy of CQE in treating human OA.

Looking ahead, future studies are needed to evaluate the sustained effects of CQE on OA and potential long-term side effects. Human clinical trials will be essential to translate these findings into clinical practice, assessing the safety, efficacy, and optimal dosing of CQE in patients with OA. Additional mechanistic studies are required to elucidate how CQE and its biologically active components interact with cellular pathways involved in inflammation, cartilage degradation, and bone remodeling. Comparative studies with standard OA treatments would also help position CQE within the therapeutic landscape. Moreover, exploring the potential synergistic effects of CQE when combined with other natural compounds could lead to the development of more effective multi-component therapies. Dose optimization studies and the identification of specific biomarkers associated with CQE’s efficacy could enhance treatment monitoring and personalization. Finally, investigating the therapeutic benefits of CQE in related conditions such as rheumatoid arthritis or osteoporosis could expand its potential applications.

## Conclusions

This study provides compelling evidence that CQE, enriched with quercetin and isorhamnetin, exhibits significant anti-osteoarthritic effects in an MIA-induced OA rat model. CQE effectively mitigated knee joint damage, reduced subchondral bone erosion, enhanced bone volume and trabecular structure, and preserved cartilage integrity by maintaining proteoglycan content and the expression of key cartilage components such as COL2A1 and aggrecan. Additionally, CQE significantly suppressed the mRNA expression of inflammatory mediators, cytokines, and MMPs, highlighting its strong anti-inflammatory and cartilage-protective properties. The synergistic action of quercetin and isorhamnetin within CQE likely enhances its overall therapeutic potential, offering a multifaceted approach to managing OA. However, while these findings are promising, the study’s limitations, including the use of an animal model and the lack of long-term data, must be addressed. Future research should focus on validating these results through human clinical trials, exploring the long-term safety and efficacy of CQE, and further elucidating its molecular mechanisms. Additionally, investigating the potential of combining CQE with other natural compounds or standard OA treatments could optimize its therapeutic use. Ultimately, this research suggests that CQE could be a promising natural therapeutic agent for managing OA, offering a novel approach to slowing disease progression and alleviating symptoms.

## Data Availability

The data used to support this study are included with the article.
